# Species Diversity and Chemotypes of *Fusarium* Species Associated With Maize Stalk Rot in Yunnan Province of Southwest China

**DOI:** 10.3389/fmicb.2021.652062

**Published:** 2021-08-20

**Authors:** Kaifei Xi, Liuying Shan, Yini Yang, Guoqing Zhang, Jun Zhang, Wei Guo

**Affiliations:** ^1^Institute of Food Science and Technology, Chinese Academy of Agricultural Sciences, Beijing, China; ^2^Key Laboratory of Agro-Products Quality and Safety Control in Storage and Transport Process, Ministry of Agriculture and Rural Affairs, Beijing, China; ^3^The Central Agricultural Broadcasting and Television School, Beijing, China; ^4^General Office of the Ministry of Agriculture and Rural Affairs, Beijing, China

**Keywords:** maize stalk rot, *Fusarium* spp., diversity, pathogenicity, chemotypes

## Abstract

Maize stalk rot caused by *Fusarium* species is one of the most important fungal diseases of maize throughout the world. The disease is responsible for considerable yield losses and has also been associated with mycotoxin contamination of the crop. In this study, a survey of maize stalk rot was performed in seven locations of Yunnan Province in China during the cropping season of 2015 and 2016. Based on morphological and molecular characteristics, 204 isolates belonging to 12 *Fusarium* spp. from symptomatic stalks of maize were identified. Among the isolated strains, 83 were identified as *Fusarium meridionale* (40.5%), 46 as *Fusarium boothii* (22.5%), 34 as *Fusarium temperatum* (16.5%), 12 as *Fusarium equiseti* (5.9%), 10 as *Fusarium asiaticum* (4.9%), six as *Fusarium proliferatum* (3.0%), four as *Fusarium verticillioides* (2.0%), four as *Fusarium incarnatum* (2.0%), two as *Fusarium avenaceum* (1.0%), one as *Fusarium cerealis* (0.5%), one as *Fusarium graminearum* (0.5%), and one as *Fusarium cortaderiae* (0.5%). *Fusarium cortaderiae* was the first report on the causal agent of maize stalk rot disease in China. These isolates were divided into five chemotypes: nivalenol (NIV), deoxynivalenol (DON), beauvericin (BEA), zearalenone (ZEN), and fumonisin (FUM). Phylogenetic analysis based on partial sequences of the translation elongation factor 1α (*TEF1-α*) showed a high degree of interspecific polymorphisms among the isolates. Pathogenicity analysis on maize stalks indicated that all the 12 species of *Fusarium* were able to cause the disease symptoms with different aggressiveness. This study on population, pathogenicity, and toxigenic chemotypes of *Fusarium* species associated with maize stalk rot in Yunnan Province of southwest China, will help design an effective integrated control strategy for this disease.

## Introduction

In the Yunnan Province of southwest China, maize plays a crucial role in local agricultural production. In this region, the maize crop’s yield and quality are particularly affected by stalk rot diseases caused by *Fusarium* species. *Fusarium* is an important plant pathogenic fungus with a wide range of hosts, including corn, wheat, rice, and other cereal crops (Boutigny et al., 2011). These pathogens cause ear and stalk rot disease, potentially damaging to crop yield and food safety. Different *Fusarium* species can produce toxic chemicals known as mycotoxins, which can be an important risk to both animal and human health if accumulated to an unsafe level (Sampietro et al., 2012; Kuhnem et al., 2016).

*Fusarium* genus has numerous species, which are morphologically indistinguishable, so they are very difficult to identify at the species level (Thomas et al., 2019). *Fusarium graminearum* species complex (FGSC) has been divided into biogeographically distinct lineages consisting of at least 16 species. Members of the FGSC are also classified into the broader *Fusarium sambucinum* species complex (FSAMSC; Starkey et al., 2007; O’Donnell et al., 2008, 2013; Sarver et al., 2011). Various members of FGSC show different geographic distribution and host preferences (Lee et al., 2015). Among different species in FGSC, *F. graminearum* is considered as an important pathogen of maize (Moreno-González et al., 2004). Earlier studies have reported that *F. graminearum* could cause seedling blight and root rot (Du et al., 1997; Munkvold and O’Mara, 2002). However, a previous study reported the presence of *Fusarium culmorum*, *Fusarium solani*, *Fusarium semitectum*, *Fusarium verticillioides*, and *F. graminearum* from the lodged maize plants (Ares et al., 2004). Another study showed that *F. graminearum* was the most aggressive strain during pathogenicity tests on maize (Lamprecht et al., 2011). In addition, *F. graminearum* is a dominant pathogen associated with *Fusarium* head blight (FHB) in North America and Europe (O’Donnell et al., 2004; Starkey et al., 2007), whereas *Fusarium asiaticum* has been found as a major species in Asia (Qiu et al., 2014). *Fusarium graminearum* is often found on wheat, but *Fusarium boothii* and *Fusarium meridionale* are frequent pathogens of maize, and *Fusarium asiaticum* is commonly reported from rice (Maier et al., 2006). Besides, *F. verticillioides* is one of the most common pathogens causing ear and stalk rot in maize. This species is widespread in areas with relatively warm and dry weather (Czembor et al., 2019), including the European and the Kansas state of the United States. In China, many *Fusarium* species are associated with ear and stalk rot diseases of maize, which resulted in significant yield losses and mycotoxin contamination problems. In China, the notable *Fusarium* species isolated from maize are *F. verticillioides*, *F. graminearum*, *F. meridionale*, and *Fusarium temperatum* (Duan et al., 2016). *Fusarium temperatum* is also an important maize pathogen and described as a new species causing disease in maize crop (Scauflaire et al., 2011). These pathogens can produce different toxigenic chemotypes, demonstrating the tremendous potential of this species for mycotoxin contamination (Duan et al., 2016). Moreover, isolates of *F. asiaticum*, isolated from head blight infected wheat plants produced 3A-deoxynivalenol (DON) or nivalenol (NIV). It was also showed that the isolates producing different mycotoxins also have differences in growth rate, pathogenicity, conidial length, fecundity, trichothecene accumulation and showed a varying degree of resistance to benzimidazole (Zhang et al., 2012). Although, many *Fusarium* species have been reported to responsible for maize ear and stalk rot disease in China, no detailed studies have been done in Yunnan Province based on composition, pathogenicity, and toxigenic chemotypes.

As the largest grain crop in Yunnan Province, maize is distributed throughout the province. Yunnan has diverse environmental conditions and topography, where the maize planted in areas have temperature ranging from 9 to 30°C and altitude ranging from 700 to 2,400 m. So, climatic conditions, soil type, water availability, farming system, and planting habits vary significantly throughout the province. Besides, different maize varieties planted in different parts of Yunnan have various growth characteristics, and the yield also varies considerably among the different areas of the province. In this study, diseased stalks of maize were collected from seven locations of Yunnan Province during the cropping season of 2015 and 2016. The study aimed to determine species diversity, pathogenicity, and toxigenic chemotypes of the *Fusarium* species causing maize stalk rot in Yunnan Province to design an effective integrated control strategy for this disease.

## Materials and Methods

### Fungal Isolation, Purification, and Morphological Characterization

**Figure 1:**
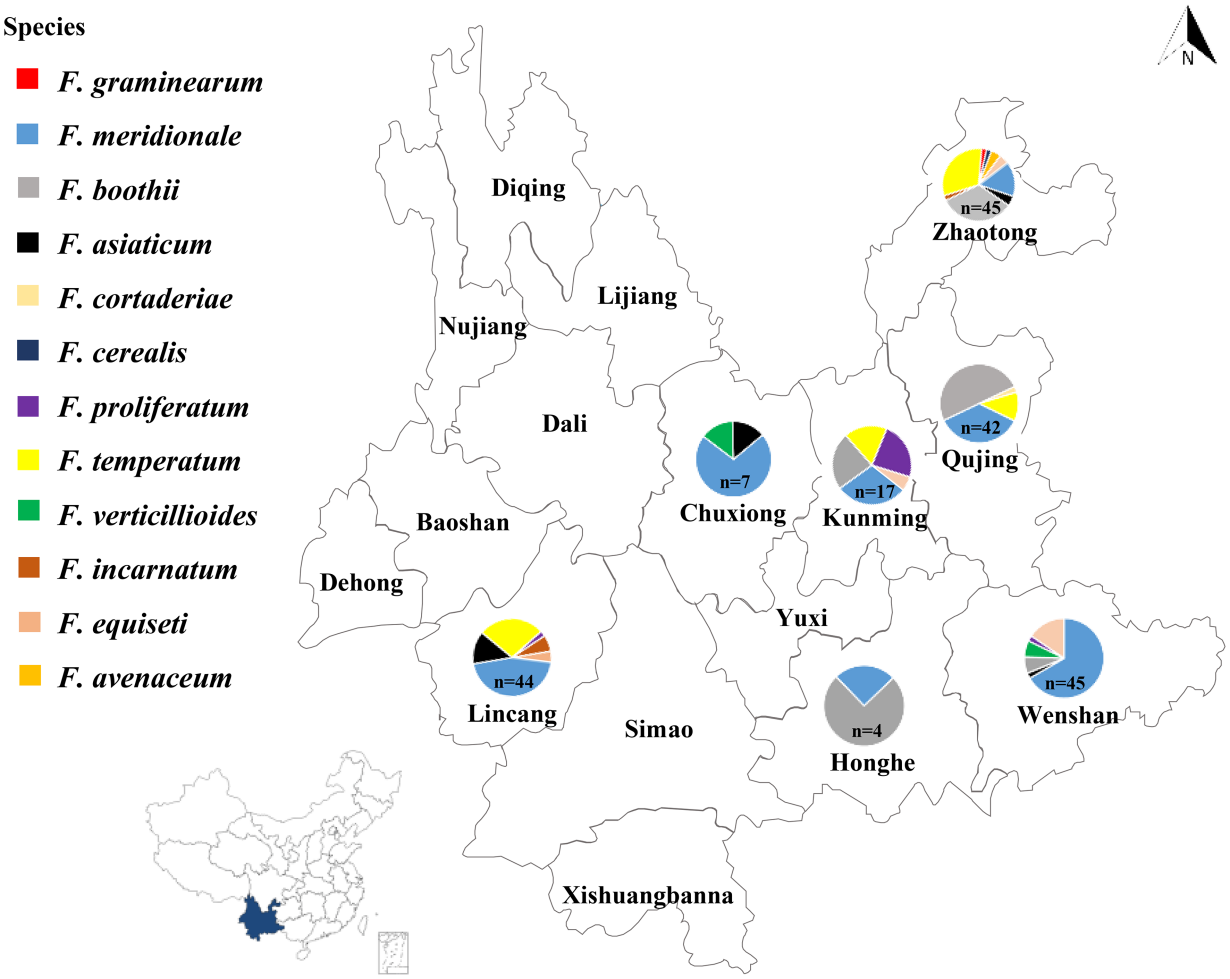
Geographical distribution of *Fusarium* species isolated from maize stalks in Yunnan Province of China. The number of *Fusarium* isolates collected in each location was indicated by *n*: Wenshan (*n* = 45), Honghe (*n* = 4), Lincang (*n* = 44), Chuxiong (*n* = 7), Kunming (*n* = 17), Qujing (*n* = 42), and Zhaotong (*n* = 45).

Stalks of maize showing typical rot symptoms were collected from seven maize-planting locations in Yunnan Province of China during the cropping season of 2015 and 2016 (Figure 1). The diseased samples were cut into small pieces (approximately 5 mm^2^) and soaked in 75% ethanol for 2 min. Subsequently, washed three times with sterile water and dried using autoclaved tissue towels. Later, the samples were placed onto potato dextrose agar (PDA) plates, which were supplemented with streptomycin sulfate (150 μg/ml) and kanamycin (150 μg/ml). The PDA plates were incubated at 25°C for 2–3 days in darkness. Fungal colonies showing various morphological features were selected. Fungal isolates were grown on PDA after single spore purification by following the procedure described by Xi et al. (2019). Morphological features of the fungal isolates were observed on PDA and carnation leaf agar (CLA). The appearance of the fungal colonies was recorded after the mycelium fully covered the whole PDA plate. Six *Fusarium* species including *Fusarium avenaceum*, *Fusarium cerealis*, *Fusarium equiseti*, *F. graminearum*, *F. proliferatum*, and *F. verticillioides* were confirmed by following the details mentioned in The *Fusarium* Laboratory Manual (Leslie and Summerell, 2006). For the identification of the other six species such as *F. meridionale*, *F. boothii*, *F. temperatum*, *F. asiaticum*, *Fusarium incarnatum*, and *Fusarium cortaderiae*, recently published materials were followed (Lamprecht et al., 2011; Scauflaire et al., 2011; Castañares et al., 2016; Walkowiak et al., 2016; Avila et al., 2019). The size of microconidia and macroconidia were taken as average from 50 measurements of each isolate.

### Species and Chemotype Determination

About 10 mm mycelial plugs from the colony’s edge were inoculated to CM liquid medium and incubated in a shaker without light (175 rpm, 25°C) for 5 days. After incubation, the mycelia were collected by centrifugation (4,000 rpm, 5 min) and stored at −80°C until the subsequent use. Total DNA was extracted using a ZR fungal DNA Kit (ZYMO Research, United States) by following the manufacturer’s instruction and stored at −20°C until the subsequent use. Sequences of the translation elongation factor 1α (*TEF-1α*) from each isolate were amplified using primers EF-1 (5'-ATGGGTAAGGARGACAAGAC-3'), and EF-2 (5'-GGARGTACCAGTSATCATGTT-3'; Geiser et al., 2004). The resulted sequences were compared with the NCBI database^1^ and *Fusarium* database (FUSARIUM-ID v.1.0 database)^2^ for species determination.

To identify each isolate’s chemotypes, six specific mycotoxin-producing genes were amplified by PCR using specific primers as previous described (Ward et al., 2002; Jennings et al., 2004; Kulik et al., 2007; Meng et al., 2010; Duan et al., 2016). The sequence of primers used to amplify these genes has been mentioned in Supplementary Table S1. The PCR was done in a 20 μl reaction mixtures including 1 μl of template DNA, 10 μl of 2× DreamTaq PCR Mix (Thermo Fisher Scientific, United States), 7 μl of sterile water, and 1 μl of each primer (10 μM). Amplification reactions were carried out in a C1000 Touch thermal cycler (Applied Biosystems, BIO-RAD, United States).

### Phylogenetic Analysis of the *TEF-1α* Gene Sequence Data

The *TEF1-α* gene always appeared to have a single copy in *Fusarium* and showed high levels of sequence polymorphism in closely related species (Geiser et al., 2004). All of the sequences (*n* = 204) were aligned online using the MAFFT alignment program (Katoh and Standley, 2013). Alignments were adjusted manually using Clustal X (Thompson et al., 1994). A phylogenetic tree from multiple alignments of the 204 sequences was constructed using the neighbor-joining method calculated with MEGA X (Sudhir et al., 2018). The Interactive Tree of Life^3^ was used to beautify the phylogenetic tree. Clade support was inferred from 1,000 bootstrap replicates.

### Pathogenicity Tests on Maize Stalks

B73 maize plants were inoculated at the 10-leaves stage by punching a hole in the stalk at the second or third internode above the soil line using a sterile toothpick. Then 20 μl conidia suspension was injected from representative isolates at a concentration of 10^6^/ml. Mock-inoculated maize stalks were treated with sterilized water. The inoculation site was wrapped using a piece of sterilized gauze to conserve moisture and avoid any contamination. Each representative isolate and control were inoculated on three plants. After 7 days post-inoculation (dpi), the stalks of inoculated plants were split along the longitudinal direction for symptom measurements. The longitudinal brown infected areas were measured as the necrosis area to calculate each identified *Fusarium* species’ virulence using ImageJ software (Zhang et al., 2016).

## Results

### Isolation and Morphological Identification of *Fusarium* Species

Based on the morphological and molecular characteristics, 204 isolates were identified from seven major maize producing regions of Yunnan Province (Figure 1). Twelve *Fusarium* species were found including, 83 isolates as *F. meridionale* (40.5%), 46 isolates were *F. boothii* (22.5%), 34 isolates identified as *F. temperatum* (16.5%), 12 as *F. equiseti* (5.9%), 10 isolates were *F. asiaticum* (4.9%), six were *F. proliferatum* (3.0%), four as *F. verticillioides* (2.0%), four as *F. incarnatum* (2.0%), two as *F. avenaceum* (1.0%), one as *F. cerealis* (0.5%), one as *F. graminearum* (0.5%), and one as *F. cortaderiae* (0.5%; Figure 1).

**Figure 2:**
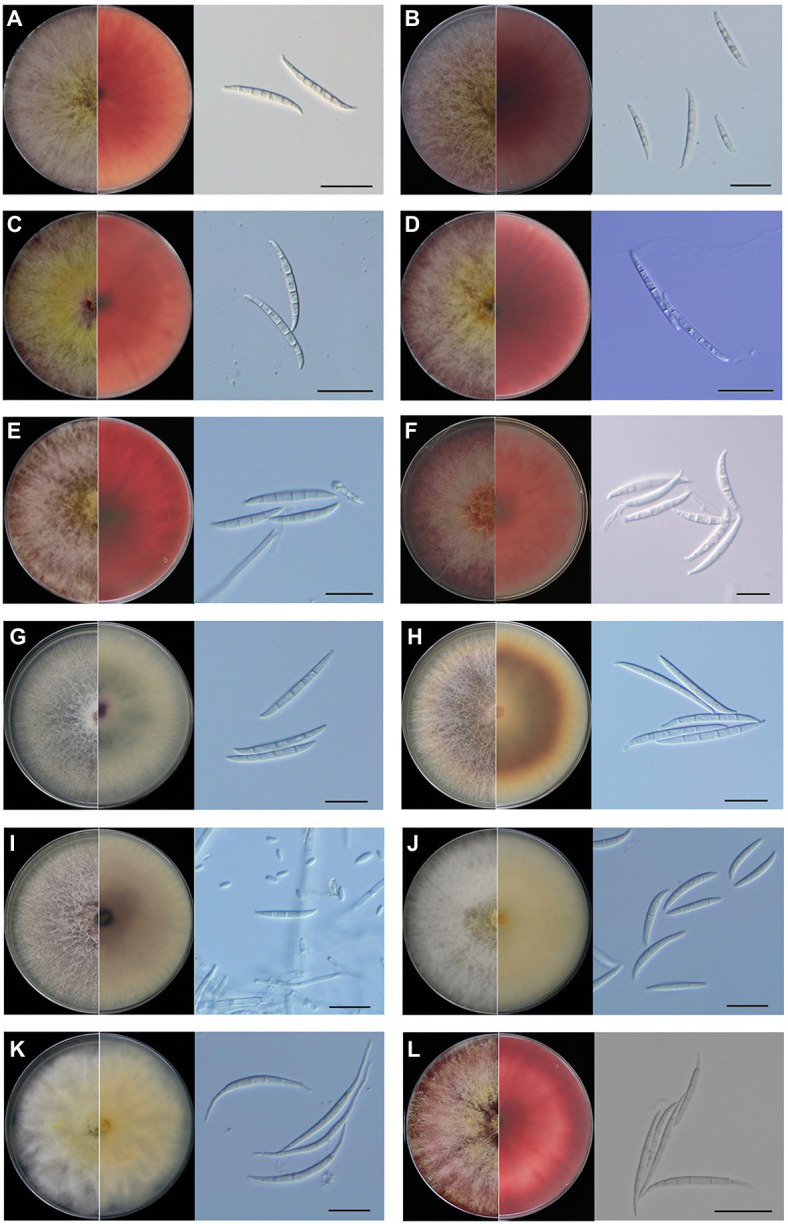
Morphological characteristics of *Fusarium* species isolated from maize stalk rot disease in Yunnan Province, China. Typical colonies of the representative *Fusarium* isolates were observed on potato dextrose agar (PDA) plates at 5 days post-inoculation (dpi). Microscopic features of macroconidia of each *Fusarium* species. **(A)** *Fusarium graminearum*, **(B)** *Fusarium meridionale*, **(C)** *Fusarium boothii*, **(D)** *Fusarium asiaticum*, **(E)** *Fusarium cortaderiae*, **(F)** *Fusarium cerealis*, **(G)** *Fusarium proliferatum*, **(H)** *Fusarium temperatum*, **(I)** *Fusarium verticillioides*, **(J)** *Fusarium incarnatum*, **(K)** *Fusarium equiseti*, and **(L)** *Fusarium avenaceum*. Scale bar = 20 μm.

All of the 12 species showed typical *Fusarium* morphological characteristics, which were consistent with the previous reports. *Fusarium graminearum*, *F. meridionale*, *F. boothii*, *F. asiaticum*, *F. cortaderiae*, and *F. cerealis* belonged to the FSAMSC and shared similar morphological characteristics. They had woolly aerial hyphae and formed red pigment in the PDA plates. At later stages, yellow hyphae were produced in the center of the colony, and the bottom of the plates became dark-red to black-red (Figures 2A–F). Mycelial growth rates of the members of FSAMSC were faster than the other six *Fusarium* species, and the *F. graminearum* showed the fastest growth rate. All members of this complex produced macroconidia, but no microconidia were observed. Macroconidia were curved at the base and apex and usually contained three or five septa. The average sizes of macroconidia (*N* = 50) were 35.9–72.7 μm long × 3.6–5.6 μm wide, 32.5–68.6 μm long × 2.2–4.4 μm wide, 33.8–61.2 μm long × 2.0–4.0 μm wide, 37.3–69.7 μm long × 2.7–5.3 μm wide, 31.7–66.3 μm long × 3.5–5.5 μm wide, and 25.8–55.9 μm long × 3.0–7.0 μm wide for *F. graminearum*, *F. meridionale*, *F. boothii*, *F. asiaticum*, *F. cortaderiae*, and *F. cerealis*, respectively (Figure 2; Supplementary Table S2).

*Fusarium proliferatum*, *F. temperatum*, and *F. verticillioides* belonged to *Fusarium fujikuroi* species complex (FFSC) and shared similar morphological characteristics (Figures 2G–I). They had woolly aerial hyphae, and the colonies showed spider-web like arrangement but produced different pigments. *Fusarium proliferatum* was white or light purple in the early stages. Later, it became dark purple or grayish purple (Figure 2G). *Fusarium temperatum* was light orange at the start of the growth. It later became dark purple (Figure 2H). *Fusarium verticillioides* was initially white or light purple. At the later stages, it became dark purple (Figure 2I). Compared to the species of FSAMSC, mycelial growth rates of *F. proliferatum*, *F. temperatum*, and *F. verticillioides* were slow. They could produce macroconidia and microconidia on CLA. The macroconidia of *F. proliferatum* were sickle-shaped, straight, and slender, 3–5 septa, and 35.5–55.5 μm long × 2.5–4.5 μm wide (Figure 2G). The microconidia of *F. proliferatum* were ovate or mallet, usually aseptate, concentric or pseudo cephalic, and 5.0–16.7 μm long × 1.8–3.5 μm wide. The macroconidia of *F. temperatum* were slender, mainly possessed four septa, and 26.0–67.5 μm long × 3.6–5.0 μm wide (Figure 2H). The microconidia of *F. temperatum* were long elliptic, pseudo cephalic, 0–1 septum, and 5.5–16.5 μm long × 2.0–4.0 μm wide. The macroconidia of *F. verticillioides* were sickle-shaped, straight, and slender, 3–5 septa, and 30.4–52.3 μm long × 2.6–4.0 μm wide (Figure 2I). The microconidia of *F. verticillioides* were clubbed shaped, 0–1 septum, and were 4.4–11.1 μm long × 1.5–3.7 μm wide.

*Fusarium incarnatum* and *F. equiseti* belonged to *Fusarium incarnatum*-*equiseti* species complex (FIESC) and exhibited similar morphology. Colony appearance of *F. incarnatum* and *F. equiseti* was abundant mycelium that initially white but became yellowish-brown with age (Figures 2J,K). *Fusarium incarnatum* produces straight to slightly curved macroconidia without obvious foot-shaped base cells (Figure 2J). The species also produces abundant microconidia. There was no apparent boundary between macroconidia and microconidia of *F. incarnatum*, possessed 3–5 septa, 27.5–40.5 μm long × 3.5–5.5 μm wide (Figure 2J). However, *F. equiseti* only produces macroconidia. The macroconidia of *F. equiseti* were sickle-shaped, slender, and curved, apical cells slender and a prominent heel, generally 3–6 septa, and 35.5–60.0 μm long × 3.0–5.0 μm wide (Figure 2K). *Fusarium avenaceum* belonged to *Fusarium tricinctum* species complex (FTSC). On PDA plates, aerial mycelia were compact and woolly, having white to light yellow color with central spore mass pale orange to brown and the colony reverse was carmine (Figure 2L). The macroconidia of *F. avenaceum* were slender and straight, linear, 4–6 septa, and 45.5–65.5 μm long × 3.5–4.5 μm wide (Figure 2L). Microconidia were fusoid, 1–2 septa, and ranged from 13.4–24.6 μm long × 2.6–4.8 μm wide. The morphological details of the colony, macroconidia and microconidia of these *Fusarium* species and the growth diameter of each *Fusarium* colony on PDA plates after 3 days has also been shown in Supplementary Table S2.

### Analysis of Toxigenic Chemotypes

In FGSC, the *Tri* genes cluster is responsible for the production of different types of toxins. Three primers based on *Tri3*, *Tri7*, and *Tri8* intergenic sequences, Tri315F/R, nivPF/R, and MinusTri7F/R, were used to amplify specific 15-AcDON fragments of 864 bp, NIV fragments of 450 bp, and 3-AcDON fragments of 483 bp, respectively. Similarly, the *FUM1* gene was used to detect the Fumonisins (FBs) with a fragment of 750 bp. Whereas the *esyn1* gene was used to detect the beauvericin (BEA) with a fragment of 600 bp. Also, the PKS4 gene was used to detect the zearalenone (ZEN) with a fragment of 280 bp.

**Figure 3:**
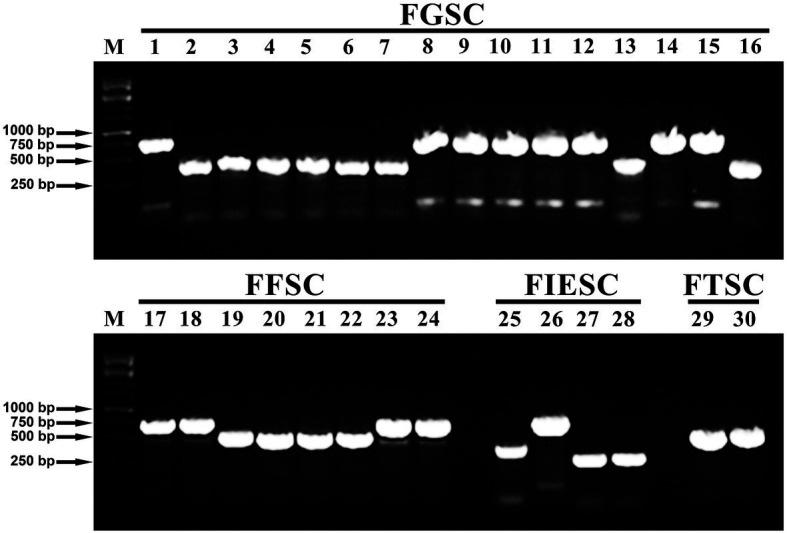
Visualization of PCR-based chemotype analysis from all of the 12 *Fusarium* species using representative isolates. Lane M: Marker; Lanes 1: detection of 15Ac-deoxynivalenol (DON) chemotypes produced by the *F. graminearum* isolate (YNF16-37); Lanes 2–7: detection of nivalenol (NIV) chemotypes produced by *F. meridionale* isolates (YNF15-50, YNF15-21, YNF15-78, YNF15-29, YNF16-19, and YNF16-55); Lanes 8–12: detection of 15Ac-DON chemotypes produced by *F. boothii* isolates (YNF15-23, YNF15-40, YNF16-17, YNF16-68, and YNF16-113); Lanes 13: detection of NIV chemotypes produced by the *Fusarium asiaticum* isolate (YNF15-56); Lanes 14: detection of 15Ac-DON chemotypes produced by the *F. asiaticum* isolate (YNF15-59); Lanes 15: detection of 15Ac-DON chemotypes produced by the *Fusarium cortaderiae* isolate (YNF16-101); Lanes 16: detection of NIV chemotypes produced by the *F. cerealis* isolate (YNF16-22); Lanes 17–18: detection of fumonisin (FUM) chemotypes produced by *F. proliferatum* isolates (YNF15-10 and YNF16-118); Lanes 19–22: detection of beauvericin (BEA) chemotypes produced by *F. temperatum* isolates (YNF15-67, YNF16-04, YNF16-77, and YNF16-116); Lanes 23–24: detection of FUM chemotypes produced by *F. verticillioides* isolates (YNF15-04 and YNF15-98); Lanes 25: detection of 3Ac-DON chemotypes produced by the *F. incarnatum* isolate (YNF15-87); Lanes 26: detection of 15Ac-DON chemotypes produced by the *F. incarnatum* isolate (YNF15-93); Lanes 27–28: detection of zearalenone (ZEN) chemotypes produced by *F. equiseti* isolates (YNF15-01 and YNF15-64); Lanes 29–30: detection of BEA chemotypes produced by *F. avenaceum* isolates (YNF16-15 and YNF16-14).

The PCR amplification results showed that all of the 12 *Fusarium* species can synthesize mycotoxins and the amplification results of the representative isolates were shown in Figure 3. Among all of 204 isolates, 53 isolates produced the DON chemotype, 93 isolates had the NIV chemotype, 10 isolates potentially produced FB1, 12 isolates had ZEN chemotype, and 36 isolates potentially produced BEA. Interestingly, all of *F. cerealis* and *F. meridionale* isolates produced the NIV chemotype. Similarly, the chemotypes of *F. boothii*, *F. cortaderiae*, *F. incarnatum*, and *F. graminearum* were categorized as the DON chemotype. Among the DON-producing isolates, all of *F. boothii*, *F. cortaderiae*, and *F. graminearum* isolates potentially produced 15-AcDON. However, one *F. incarnatum* isolate possessed the 15-AcDON chemotypes, the other three isolates represented the 3-AcDON chemotype. Conversely, all *F. equiseti* isolates were the ZEN chemotype, although *F. incarnatum* and *F. equiseti* belonged to the same species complexes. Also, most of *F. asiaticum* isolates mainly belonged to the NIV chemotype, and only one was categorized as the 15-AcDON chemotype. All of the isolates of *F. verticillioides* and *F. proliferatum* were the fumonisin chemotype. However, all of *F. temperatum* isolates like *F. avenaceum* isolates were the BEA chemotype (Table 1).

**Table 1:** Chemotypes of *Fusarium* species identified in this study.

Species	Percentage (%)	Number	Toxigenic chemotypes				
			NIV	DON	BEA	FUM	ZEN
*Fusarium asiaticum*	5	10	9	1	0	0	0
*Fusarium boothii*	22.5	46	0	46	0	0	0
*Fusarium cortaderiae*	0.5	1	0	1	0	0	0
*Fusarium graminearum*	0.5	1	0	1	0	0	0
*Fusarium meridionale*	40.5	83	83	0	0	0	0
*Fusarium cerealis*	0.5	1	1	0	0	0	0
*Fusarium verticillioides*	2	4	0	0	0	4	0
*Fusarium proliferatum*	3	6	0	0	0	6	0
*Fusarium temperatum*	16.5	34	0	0	34	0	0
*Fusarium equiseti*	6	12	0	0	0	0	12
*Fusarium incarnatum*	2	4	0	4	0	0	0
*Fusarium avenaceum*	1	2	0	0	2	0	0
Total	100	204	93	53	36	10	12

### Phylogenetic Analysis Based on the Partial *TEF-1α* Sequences

**Figure 4:**
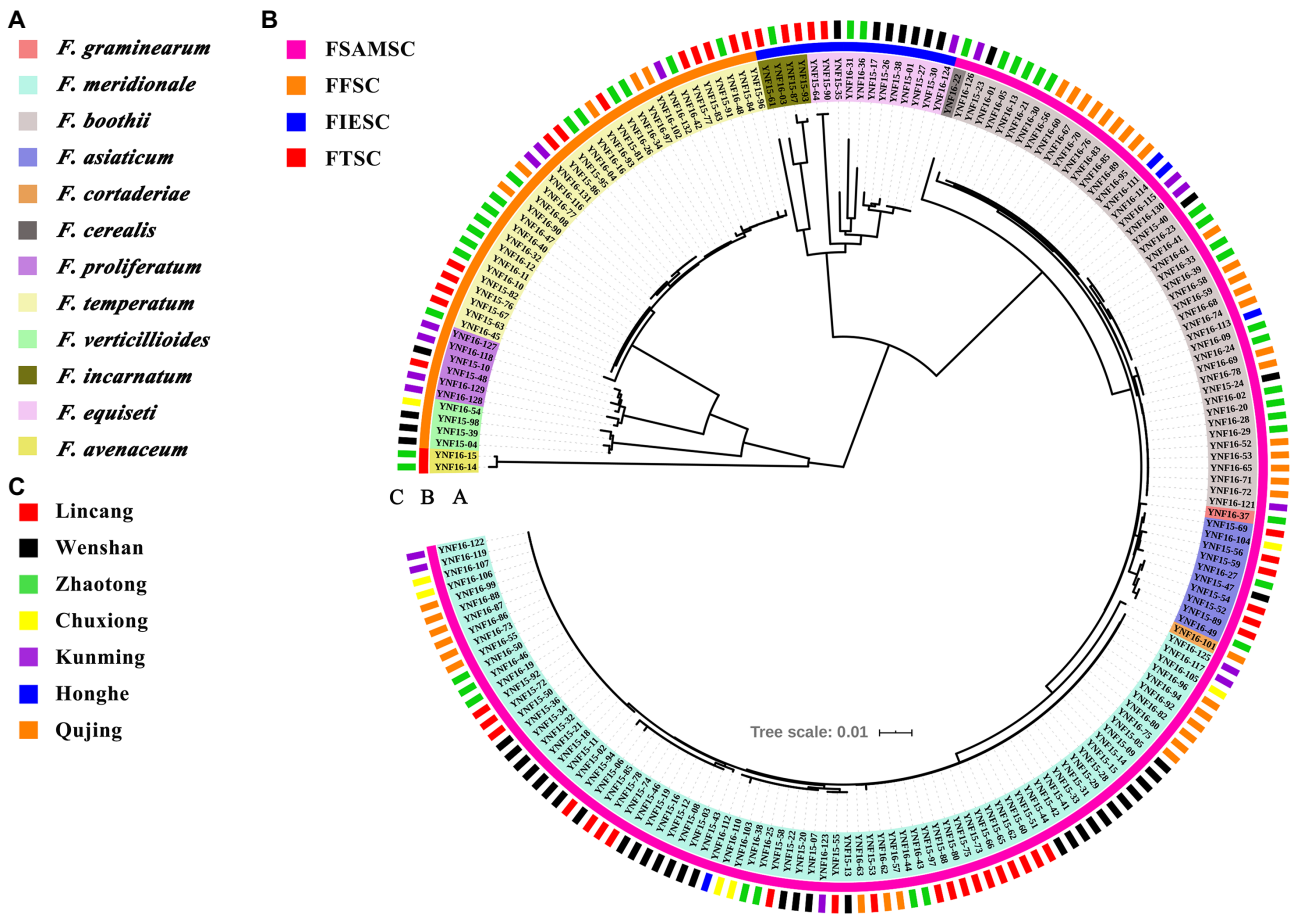
Phylogenetic analysis of *Fusarium* species associated with maize stalk rot disease in Yunnan Province of China. The tree was constructed by the neighbor-joining method based on partial translation elongation factor 1α (*TEF-1α*) sequences. Clade support was inferred from 1,000 bootstrap replicates. The Interactive Tree of Life (see footnote 3) was used to beautify the phylogenetic tree. **(A)** Species information about each *Fusarium* isolates identified in this study and their corresponding color information. **(B)** The abbreviation of *Fusarium* species complexes. FSAMSC means *Fusarium sambucinum* species complex. FFSC means *Fusarium fujikuroi* species complex. FIESC means *Fusarium incarnatum*-*equiseti* species complex. FTSC means *Fusarium tricinctum* species complex. **(C)** The geographical location of each *Fusarium* isolates identified in this study.

For phylogenetic analysis, a neighbor-joining tree was constructed using the partial *TEF-1α* gene sequences, including all isolates in this study (Figure 4). The GenBank accession numbers for the *TEF-1α* gene sequences of all the 204 strains are listed in Supplementary Table S3. The phylogenetic analysis showed that isolates of *F. graminearum*, *F. meridionale*, *F. boothii*, *F. asiaticum*, *F. cortaderiae*, and *F. cerealis* belonging to the FSAMSC were clustered into one big branch. It is worth noting that the isolates of *F. asiaticum*, *F. boothii*, *F. cortaderiae*, *F. graminearum*, and *F. meridional* showed a closer phylogenetic relationship compared to the isolate of *F. cerealis* because these isolates belonged to the FGSC, which is a part of FSAMSC. The isolates of *F. proliferatum*, *F. temperatum*, and *F. verticillioides* formed an independent branch in the phylogenetic tree owing to these isolates belonging to the FFSC. Similarly, isolates of *F. incarnatum* and *F. equiseti* formed an independent branch because of these isolates belonging to the FIESC. Likewise, isolates of *F. avenaceum* classified into FTSC showed another independent branch in the tree (Figure 4). These results indicated that isolates of *Fusarium* species showed a high degree of interspecific polymorphisms variation and was unrelated to geographic distribution.

### Pathogenicity Tests on Maize Stalks

**Figure 5:**
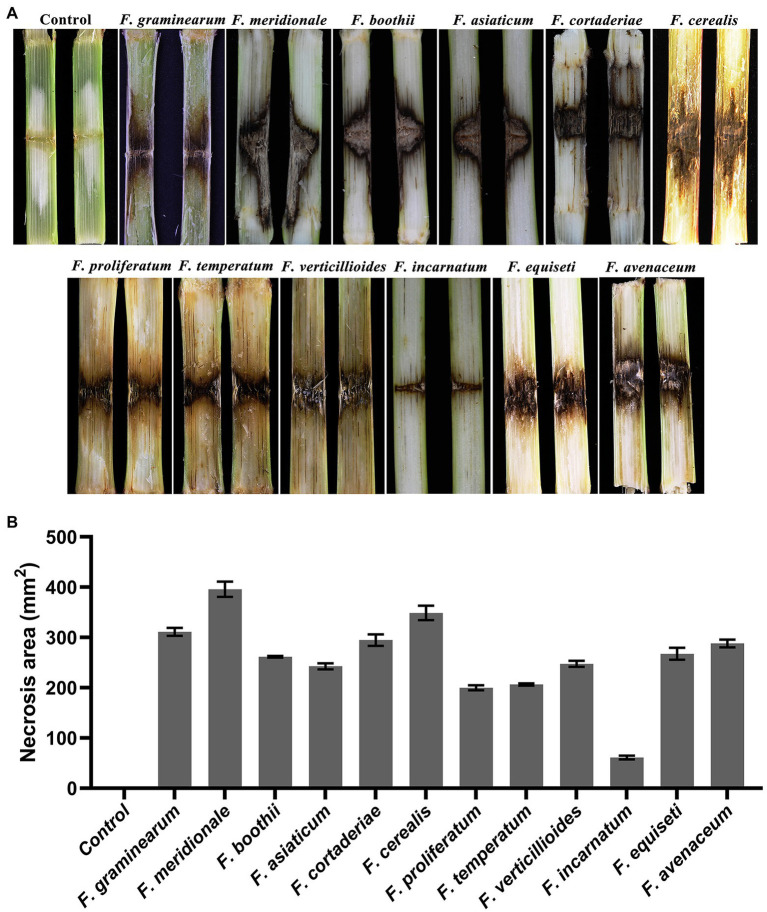
Symptoms on maize stalks inoculated with representative *Fusarium* isolates. **(A)** Symptoms of maize stalks inoculated with representative *Fusarium* isolate at 7 dpi. No symptoms were observed in the control plants. **(B)** Statistical analysis of necrotic areas around the insertion point inoculated with representative *Fusarium* isolates at 7 dpi. The longitudinal brown infected areas were used to evaluate the virulence of each identified *Fusarium* species. Each assay was performed on three independent biological repeats.

To test the pathogenicity of the 12 isolated *Fusarium* species, the stalks of B73 maize plants at the 10-leaf stage were inoculated with each representative fungal species. The symptoms and severity of the disease were recorded at the 7 dpi. The results showed that all of the *Fusarium* species are pathogenic to maize stalks and showed distinct discoloration of internal stalk tissues around the inoculation site (Figure 5A). The longitudinal brown infected areas of maize stalks were measured to evaluate the virulence of each identified *Fusarium* species (Figure 5B). The results indicated that isolates of *F. meridionale* are the most aggressive among all of the isolates. Pathogenicity of these isolates was confirmed by reisolating the fungus from symptomatic tissues but not from the control plants.

## Discussion

*Fusarium* spp. can cause various diseases at different growth stages of maize, such as root, seedling, stalk, and ear rot, leading to yield losses, and reduction of grain quality (Pfordt et al., 2020). Most of *Fusarium* spp. can produce different mycotoxins to contaminate small grain crops from pre-harvest to post-harvest stages. The gene of *TEF1-α* always appeared to have a single copy in *Fusarium*. It showed high levels of sequence polymorphism in closely related species, and the DNA sequence based on the *TEF1-α* was often used to identify the putative *Fusarium* species (Geiser et al., 2004; Berruezo et al., 2018; Fang et al., 2020; Wang et al., 2021). In this study, 12 *Fusarium* species were isolated and identified from symptomatic maize stalks based on morphological characteristics, phylogenetic analysis (*TEF1-α*), and Koch’s postulates. Among them, *F. meridionale* (40.5%), *F. boothii* (22.5%), and *F. temperatum* (16.5%) were more prevalent. For the consecutive two cropping seasons of 2015 and 2016, we find the *F. meridionale*, *F. boothii*, *F. asiaticum*, *F. proliferatum*, *F. temperatum*, *F. verticillioides*, *F. incarnatum*, and *F. equiseti* from various locations of the Yunnan Province. Previous studies reported *F. meridionale*, *F. graminearum*, and *F. cortaderiae* were found from diseased maize plants in Brazil, and *F. meridionale* was the dominant (Kuhnem et al., 2016). Our results showed that *F. meridionale* was distributed throughout the seven-sampling locations and was also the major pathogen causing maize stalk rot disease in Yunnan Province of China. These results indicate that the environmental conditions were suitable for *F. meridionale* in Yunnan Province. However, only two isolates were individually identified as *F. graminearum* and *F. cortaderiae* in the present study. *Fusarium cortaderiae* was identified for the first time from maize in China. Previous study on the pathogenic *Fusarium* spp. causing maize ear rot in China showed that *F. verticillioides*, *F. graminearum*, *F. meridionale*, and *F. boothii* were dominant *Fusarium* species (Duan et al., 2016). However, only a few samples were taken from Yunnan Province in that study, which cannot represent the diversity of *Fusarium* spp. associated with maize ear rot disease in the whole province. In contrast, present studies focused on providing useful information of dominant pathogenic *Fusarium* species and their potential mycotoxins associated with maize in Yunnan Province. In this study, we found that *F. meridionale* and *F. boothii* were prevalent to cause maize stalk rot. However, the percentage of *F. verticillioides* and *F. graminearum* in this survey was unexpected. Notably, *F. temperatum*, a new species recently separated from *Fusarium subglutinans*, need to pay more attention in the future. *Fusarium* species can infect the stalk during the whole vegetation period by systemic spread after colonization of the roots (Murillo-Williams and Munkvold, 2008), through young leaf sheaths, by seed transmission and *via* wounds caused by hail or insect feeding (Gai et al., 2018). Stalk rot of maize results in defective grain filling, premature senescence, and lodging, which negatively affects production, harvesting, and yield (Quesada-Ocampo et al., 2016). In the present study, the pathogenicity tests showed that all of the *Fusarium* isolates could cause severe symptoms of maize stalk rot, but the extent of lesion spread was different. *Fusarium meridionale* was the most aggressive species to infect maize stalks. So, we have reasons to believe that differences in compositions of *Fusarium* spp. associated with maize stalk rot disease in Yunnan Province were caused by local climatic conditions.

To investigate the ability to produce mycotoxins of *Fusarium* species causing maize stalk rot in Yunnan Province, the toxigenic chemotypes were also evaluated by specific PCR assays. The pathogenicity analysis showed that there was no relationship between the pathogenicity and the type of mycotoxin production. Generally, pathogenicity was not influenced by the type of mycotoxin produced (Adams and Hart, 1989; Goswami and Kistler, 2005). However, the pathogenicity of the *F. graminearum* to wheat plant has a relationship with the type of the mycotoxin (Shin et al., 2018). Another study on FSAMSC reported that the aggressiveness of the pathogen was related to the type of mycotoxin produced by the pathogen (Laraba et al., 2021). Our results indicated that 45.6% (93/204) strains were NIV producers, whereas 26.0% (53/204) stains were DON producers. So, the contaminations of NIV and DON in maize-related agro-products should be given particular attention in Yunnan Province of China. Besides, the identification of *F. cortaderiae*, *F. cerealis*, and *F. avenaceum* are reported for the first from Yunnan Province, which needs urgent attention to prevent their widespread. These results will provide useful information to design an effective strategy for the control of disease caused by *Fusarium* species in Yunnan Province of China.

## Conclusion

In the 2 years of investigation, *F. meridionale* (40.5%), *F. boothii* (22.5%), and *F. temperatum* (16.5%) were the most frequent *Fusarium* species to cause maize stalk rot disease in Yunnan Province of China. The dominance of the NIV chemotype among isolates needs to pay more attention to food safety and animal health because of the more significant toxic potential of NIV relative to DON. Besides, *F. temperatum* associated with BEA mycotoxins represented a toxigenic risk for maize production. The current results on species diversity of *Fusarium* spp. and mycotoxin contaminations associated with maize stalk rot disease will provide valuable information to design effective strategies to control the disease caused by *Fusarium* spp.

## Data Availability Statement

The datasets presented in this study can be found in online repositories. The names of the repository/repositories and accession number(s) can be found in the article/Supplementary Material.

## Author Contributions

WG conceived and designed the experiments. WG and JZ collected the samples in the field. YY and GZ provided substantial assistance to collect the samples in the field. KX and LS performed the experiments. KX and WG wrote and edited the manuscript. All authors contributed to the article and approved the submitted version.

## Conflict of Interest

The authors declare that the research was conducted in the absence of any commercial or financial relationships that could be construed as a potential conflict of interest.

## Publisher’s Note

All claims expressed in this article are solely those of the authors and do not necessarily represent those of their affiliated organizations, or those of the publisher, the editors and the reviewers. Any product that may be evaluated in this article, or claim that may be made by its manufacturer, is not guaranteed or endorsed by the publisher.

## Footnotes

^1^ https://www.ncbi.nlm.nih.gov/

^2^ http://isolate.fusariumdb.org/blast.php

^3^ https://itol.embl.de
