# Supplementary material for: Species Diversity and Chemotypes of Fusarium Species Associated With Maize Stalk Rot in Yunnan Province of Southwest China
Source: Front Microbiol. 2021 Aug 20;12:652062. doi: 10.3389/fmicb.2021.652062 (PMC8575069; doi:10.3389/fmicb.2021.652062)
Supplement: Supplementary file 1 [file Table_1.docx]

**Supplementary Table 1 | Sequences of primer pairs used to detect mycotoxin-coding genes in *Fusarium* species.**

| Mycotoxin | Gene | Primers | Primers sequence (5‘-3’) | Amplication size (bp) | References |
| --- | --- | --- | --- | --- | --- |
| Nivalenol | Tri7 | nivPF | TATCCTTGCATGGCAATGCC | 450 | Kulik et al., 2007 |
|  |  | nivPR | AAATGGCGATACGAGTATTGA |  |  |
| Fumonisin | FUM1 | Fum5F | GTCGAGTTGTTGACCACTGCG | 750 | Duan et al., 2016 |
|  |  | Fum5R | GAASGTCGCARGACCTTGTTTC |  |  |
| Beauvericin | esyn1 | esy1 | TTCAAGGGCTGGACGTCTATGTA | 600 | Kulik et al., 2007 |
|  |  | esy2 | GTGAAGAAAGCTGGCTCAACGAG |  |  |
| Zearalenone | PKS4 | F1 | CGTCTTCGAGAAGATGACAT | 280 | Menget al., 2009 |
|  |  | R1 | TGTTCTGCAAGCACTCCGA |  |  |
| 3-AcDON | MinusTri7 | F | TGGATGAATGACTTGAGTTGACA | 483 | Ward et al., 2002 |
|  |  | R | AAAGCCTTCATTCACAGCC |  |  |
| 15-AcDON | Tri315F/R | F | CTCGCTGAAGTTGGACGTAA | 864 | Jennings et al., 2004 |
|  |  | R | GTCTATGCTCTCAACGGACAAC |  |  |
