# Supplementary material for: Species Diversity and Chemotypes of Fusarium Species Associated With Maize Stalk Rot in Yunnan Province of Southwest China
Source: Front Microbiol. 2021 Aug 20;12:652062. doi: 10.3389/fmicb.2021.652062 (PMC8575069; doi:10.3389/fmicb.2021.652062)
Supplement: Supplementary file 2 [file Table_2.docx]

**Supplementary Table 2 | Morphological characteristics of colonies, macroconidia and microconidia of *Fusarium* species identified in this study**

| Species | Colony morphology | | | Macroconidia | | | Microconidia | | | |
| --- | --- | --- | --- | --- | --- | --- | --- | --- | --- | --- |
|  | Front | Back | Diameter (mm) | Width (μm) | Septum | Shape | Width (μm) | Septum | Shape | |
| *F. asiaticum* | yellowish | black red | 57-65 | 4.0±1.3 | 3-5 | Gradually curved | N.A. | N.A. | | N.A. |
| *F. boothii* | yellow | deep red | 55-67 | 3.0±1.0 | 3-5 | Gradually curved | N.A. | N.A. | | N.A. |
| *F. cortaderiae* | white and yellow | black red | 59-73 | 4.5±1.0 | 3-5 | Straight or gradually curved | N.A. | N.A. | | N.A. |
| *F. graminearum* | yellowish | deep red | 65-79 | 4.6±1.0 | 3-5 | Gradually curved | N.A. | N.A. | | N.A. |
| *F. meridionale* | white | black red | 62-77 | 3.3±1.1 | 3-5 | Gradually curved | N.A. | N.A. | | N.A. |
| *F. cerealis* | yellowish | deep red | 54-65 | 5.0±2.0 | 3-5 | Gradually curved | N.A. | N.A. | | N.A. |
| *F. verticillioides* | white and purple | deep purple | 42-47 | 3.3±0.7 | 3-5 | Straight or gradually curved | 2.6±1.1 | 0-1 | clubbed | |
| *F. proliferatum* | gray and purple | white gray | 41-43 | 3.5±1.0 | 3-5 | Gradually curved | 2.6±0.8 | 0 | ovate or mallet | |
| *F. temperatum* | white and purple | yellow gray | 37-43 | 4.3±0.7 | 3-5 | Gradually curved | 3.0±1.0 | 0-1 | long elliptic | |
| *F. equiseti* | white and yellow | yellow brown | 40-49 | 4.0±1.0 | 3-6 | Gradually curved | N.A. | N.A. | | N.A. |
| *F. incarnatum* | white and yellow | yellow brown | 46-53 | 4.5±1.0 | 3-5 | Straight or gradually curved | 4.5±1.0 | 3-5 | straight to slightly curved | |
| *F. avenaceum* | red and yellowish | black red | 22-33 | 4.0±0.5 | 4-6 | Straight or gradually curved | 3.7±1.1 | 1-2 | fusoid | |

N.A. means not available due to microconidia is absent in these *Fusarium* species.
