# Supplementary material for: Species Diversity and Chemotypes of Fusarium Species Associated With Maize Stalk Rot in Yunnan Province of Southwest China
Source: Front Microbiol. 2021 Aug 20;12:652062. doi: 10.3389/fmicb.2021.652062 (PMC8575069; doi:10.3389/fmicb.2021.652062)
Supplement: Supplementary file 3 [file Table_3.docx]

**Supplementary Table 3 | Strain information and GenBank accession numbers of each *Fusarium* isolate identified in this study.**

| **Isolate** | **Year** | **Locations** | **Species** | **Toxigenic chemotypes** | **Accession number** |
| --- | --- | --- | --- | --- | --- |
| YNF15-47 | 2015 | Wenshan | *F. asiaticum* | NIV | MT237721 |
| YNF15-52 | 2015 | Lincang | *F. asiaticum* | NIV | MT237722 |
| YNF15-54 | 2015 | Lincang | *F. asiaticum* | NIV | MT237723 |
| YNF15-56 | 2015 | Lincang | *F. asiaticum* | NIV | MT237724 |
| YNF15-59 | 2015 | Lincang | *F. asiaticum* | 15-AcDON | MT237725 |
| YNF15-69 | 2015 | Lincang | *F. asiaticum* | NIV | MT237726 |
| YNF15-89 | 2015 | Lincang | *F. asiaticum* | NIV | MT237727 |
| YNF16-27 | 2016 | Zhaotong | *F. asiaticum* | NIV | MT237728 |
| YNF16-49 | 2016 | Zhaotong | *F. asiaticum* | NIV | MT237729 |
| YNF16-104 | 2016 | Chuxiong | *F. asiaticum* | NIV | MT237730 |
| YNF15-23 | 2015 | Wenshan | *F. boothii* | 15-AcDON | MT237731 |
| YNF15-24 | 2015 | Wenshan | *F. boothii* | 15-AcDON | MT237732 |
| YNF15-40 | 2015 | Wenshan | *F. boothii* | 15-AcDON | MT237734 |
| YNF16-01 | 2016 | Zhaotong | *F. boothii* | 15-AcDON | MT237735 |
| YNF16-02 | 2016 | Zhaotong | *F. boothii* | 15-AcDON | MT237736 |
| YNF16-05 | 2016 | Zhaotong | *F. boothii* | 15-AcDON | MT237737 |
| YNF16-09 | 2016 | Zhaotong | *F. boothii* | 15-AcDON | MT237738 |
| YNF16-13 | 2016 | Zhaotong | *F. boothii* | 15-AcDON | MT237739 |
| YNF16-20 | 2016 | Zhaotong | *F. boothii* | 15-AcDON | MT237740 |
| YNF16-21 | 2016 | Zhaotong | *F. boothii* | 15-AcDON | MT237741 |
| YNF16-23 | 2016 | Zhaotong | *F. boothii* | 15-AcDON | MT237742 |
| YNF16-24 | 2016 | Zhaotong | *F. boothii* | 15-AcDON | MT237743 |
| YNF16-28 | 2016 | Zhaotong | *F. boothii* | 15-AcDON | MT237744 |
| YNF16-29 | 2016 | Zhaotong | *F. boothii* | 15-AcDON | MT237745 |
| YNF16-30 | 2016 | Zhaotong | *F. boothii* | 15-AcDON | MT237746 |
| YNF16-33 | 2016 | Zhaotong | *F. boothii* | 15-AcDON | MT237747 |
| YNF16-39 | 2016 | Zhaotong | *F. boothii* | 15-AcDON | MT237748 |
| YNF16-41 | 2016 | Zhaotong | *F. boothii* | 15-AcDON | MT237749 |
| YNF16-52 | 2016 | Qujing | *F. boothii* | 15-AcDON | MT237750 |
| YNF16-53 | 2016 | Qujing | *F. boothii* | 15-AcDON | MT237751 |
| YNF16-56 | 2016 | Qujing | *F. boothii* | 15-AcDON | MT237752 |
| YNF16-58 | 2016 | Qujing | *F. boothii* | 15-AcDON | MT237753 |
| YNF16-59 | 2016 | Qujing | *F. boothii* | 15-AcDON | MT237754 |
| YNF16-60 | 2016 | Qujing | *F. boothii* | 15-AcDON | MT237755 |
| YNF16-61 | 2016 | Qujing | *F. boothii* | 15-AcDON | MT237756 |
| YNF16-71 | 2016 | Qujing | *F. boothii* | 15-AcDON | MT237757 |
| YNF16-65 | 2016 | Qujing | *F. boothii* | 15-AcDON | MT237758 |
| YNF16-67 | 2016 | Qujing | *F. boothii* | 15-AcDON | MT237759 |

*(Continued)*

**Supplementary Table 3 | Continued**

| **Isolate** | **Year** | **Locations** | **Species** | **Toxigenic chemotypes** | **Accession number** |
| --- | --- | --- | --- | --- | --- |
| YNF16-68 | 2016 | Qujing | *F. boothii* | 15-AcDON | MT237760 |
| YNF16-69 | 2016 | Qujing | *F. boothii* | 15-AcDON | MT237761 |
| YNF16-70 | 2016 | Qujing | *F. boothii* | 15-AcDON | MT237762 |
| YNF16-72 | 2016 | Qujing | *F. boothii* | 15-AcDON | MT237763 |
| YNF16-74 | 2016 | Qujing | *F. boothii* | 15-AcDON | MT237764 |
| YNF16-76 | 2016 | Qujing | *F. boothii* | 15-AcDON | MT237765 |
| YNF16-78 | 2016 | Qujing | *F. boothii* | 15-AcDON | MT237766 |
| YNF16-83 | 2016 | Qujing | *F. boothii* | 15-AcDON | MT237767 |
| YNF16-85 | 2016 | Qujing | *F. boothii* | 15-AcDON | MT237768 |
| YNF16-89 | 2016 | Qujing | *F. boothii* | 15-AcDON | MT237769 |
| YNF16-95 | 2016 | Qujing | *F. boothii* | 15-AcDON | MT237770 |
| YNF16-111 | 2016 | Honghe | *F. boothii* | 15-AcDON | MT237771 |
| YNF16-113 | 2016 | Honghe | *F. boothii* | 15-AcDON | MT237772 |
| YNF16-114 | 2016 | Honghe | *F. boothii* | 15-AcDON | MT237773 |
| YNF16-115 | 2016 | Kunming | *F. boothii* | 15-AcDON | MT237774 |
| YNF16-121 | 2016 | Kunming | *F. boothii* | 15-AcDON | MT237776 |
| YNF16-126 | 2016 | Kunming | *F. boothii* | 15-AcDON | MT237777 |
| YNF16-130 | 2016 | Kunming | *F. boothii* | 15-AcDON | MT237778 |
| YNF16-101 | 2016 | Qujing | *F. cortaderiae* | 15-AcDON | MT237779 |
| YNF16-37 | 2016 | Zhaotong | *F.graminearum* | 15-AcDON | MT237780 |
| YNF15-02 | 2015 | Wenshan | *F. meridionale* | NIV | MT237781 |
| YNF15-03 | 2015 | Wenshan | *F. meridionale* | NIV | MT237782 |
| YNF15-05 | 2015 | Wenshan | *F. meridionale* | NIV | MT237783 |
| YNF15-06 | 2015 | Wenshan | *F. meridionale* | NIV | MT237784 |
| YNF15-07 | 2015 | Wenshan | *F. meridionale* | NIV | MT237785 |
| YNF15-08 | 2015 | Wenshan | *F. meridionale* | NIV | MT237786 |
| YNF15-09 | 2015 | Wenshan | *F. meridionale* | NIV | MT237787 |
| YNF15-11 | 2015 | Wenshan | *F. meridionale* | NIV | MT237788 |
| YNF15-12 | 2015 | Wenshan | *F. meridionale* | NIV | MT237789 |
| YNF15-13 | 2015 | Wenshan | *F. meridionale* | NIV | MT237790 |
| YNF15-14 | 2015 | Wenshan | *F. meridionale* | NIV | MT237791 |
| YNF15-15 | 2015 | Wenshan | *F. meridionale* | NIV | MT237792 |
| YNF15-16 | 2015 | Wenshan | *F. meridionale* | NIV | MT237793 |
| YNF15-18 | 2015 | Wenshan | *F. meridionale* | NIV | MT237794 |
| YNF15-19 | 2015 | Wenshan | *F. meridionale* | NIV | MT237795 |
| YNF15-20 | 2015 | Wenshan | *F. meridionale* | NIV | MT237796 |
| YNF15-21 | 2015 | Wenshan | *F. meridionale* | NIV | MT237797 |
| YNF15-22 | 2015 | Wenshan | *F. meridionale* | NIV | MT237798 |
| YNF15-28 | 2015 | Wenshan | *F. meridionale* | NIV | MT237799 |
| YNF15-29 | 2015 | Wenshan | *F. meridionale* | NIV | MT237800 |

*(Continued)*

**Supplementary Table 3 | Continued**

| **Isolate** | **Year** | **Locations** | **Species** | **Toxigenic chemotypes** | **Accession mumber** |
| --- | --- | --- | --- | --- | --- |
| YNF15-31 | 2015 | Wenshan | *F. meridionale* | NIV | MT237801 |
| YNF15-32 | 2015 | Wenshan | *F. meridionale* | NIV | MT237802 |
| YNF15-33 | 2015 | Wenshan | *F. meridionale* | NIV | MT237803 |
| YNF15-34 | 2015 | Wenshan | *F. meridionale* | NIV | MT237804 |
| YNF15-36 | 2015 | Wenshan | *F. meridionale* | NIV | MT237805 |
| YNF15-41 | 2015 | Wenshan | *F. meridionale* | NIV | MT237806 |
| YNF15-42 | 2015 | Wenshan | *F. meridionale* | NIV | MT237807 |
| YNF15-43 | 2015 | Wenshan | *F. meridionale* | NIV | MT237808 |
| YNF15-44 | 2015 | Wenshan | *F. meridionale* | NIV | MT237809 |
| YNF15-46 | 2015 | Wenshan | *F. meridionale* | NIV | MT237810 |
| YNF15-50 | 2015 | Lincang | *F. meridionale* | NIV | MT237811 |
| YNF15-51 | 2015 | Lincang | *F. meridionale* | NIV | MT237812 |
| YNF15-53 | 2015 | Lincang | *F. meridionale* | NIV | MT237813 |
| YNF15-55 | 2015 | Lincang | *F. meridionale* | NIV | MT237814 |
| YNF15-58 | 2015 | Lincang | *F. meridionale* | NIV | MT237816 |
| YNF15-60 | 2015 | Lincang | *F. meridionale* | NIV | MT237817 |
| YNF15-62 | 2015 | Lincang | *F. meridionale* | NIV | MT237818 |
| YNF15-65 | 2015 | Lincang | *F. meridionale* | NIV | MT237819 |
| YNF15-66 | 2015 | Lincang | *F. meridionale* | NIV | MT237820 |
| YNF15-72 | 2015 | Lincang | *F. meridionale* | NIV | MT237821 |
| YNF15-73 | 2015 | Lincang | *F. meridionale* | NIV | MT237822 |
| YNF15-74 | 2015 | Lincang | *F. meridionale* | NIV | MT237823 |
| YNF15-75 | 2015 | Lincang | *F. meridionale* | NIV | MT237824 |
| YNF15-78 | 2015 | Lincang | *F. meridionale* | NIV | MT237825 |
| YNF15-97 | 2015 | Lincang | *F. meridionale* | NIV | MT237826 |
| YNF15-80 | 2015 | Lincang | *F. meridionale* | NIV | MT237827 |
| YNF15-85 | 2015 | Lincang | *F. meridionale* | NIV | MT237828 |
| YNF15-88 | 2015 | Lincang | *F. meridionale* | NIV | MT237829 |
| YNF15-92 | 2015 | Lincang | *F. meridionale* | NIV | MT237830 |
| YNF15-94 | 2015 | Lincang | *F. meridionale* | NIV | MT237831 |
| YNF16-19 | 2016 | Zhaotong | *F. meridionale* | NIV | MT237832 |
| YNF16-25 | 2016 | Zhaotong | *F. meridionale* | NIV | MT237833 |
| YNF16-38 | 2016 | Zhaotong | *F. meridionale* | NIV | MT237834 |
| YNF16-43 | 2016 | Zhaotong | *F. meridionale* | NIV | MT237835 |
| YNF16-44 | 2016 | Zhaotong | *F. meridionale* | NIV | MT237836 |
| YNF16-46 | 2016 | Zhaotong | *F. meridionale* | NIV | MT237837 |
| YNF16-50 | 2016 | Zhaotong | *F. meridionale* | NIV | MT237838 |
| YNF16-55 | 2016 | Qujing | *F. meridionale* | NIV | MT237839 |
| YNF16-57 | 2016 | Qujing | *F. meridionale* | NIV | MT237840 |
| YNF16-62 | 2016 | Qujing | *F. meridionale* | NIV | MT237841 |

*(Continued)*

**Supplementary Table 3 | Continued**

| **Isolate** | **Year** | **Locations** | **Species** | **Toxigenic chemotypes** | **Accession number** |
| --- | --- | --- | --- | --- | --- |
| YNF16-63 | 2016 | Qujing | *F. meridionale* | NIV | MT237842 |
| YNF16-73 | 2016 | Qujing | *F. meridionale* | NIV | MT237843 |
| YNF16-75 | 2016 | Qujing | *F. meridionale* | NIV | MT237844 |
| YNF16-80 | 2016 | Qujing | *F. meridionale* | NIV | MT237845 |
| YNF16-82 | 2016 | Qujing | *F. meridionale* | NIV | MT237846 |
| YNF16-86 | 2016 | Qujing | *F. meridionale* | NIV | MT237847 |
| YNF16-87 | 2016 | Qujing | *F. meridionale* | NIV | MT237848 |
| YNF16-88 | 2016 | Qujing | *F. meridionale* | NIV | MT237849 |
| YNF16-92 | 2016 | Qujing | *F. meridionale* | NIV | MT237850 |
| YNF16-94 | 2016 | Qujing | *F. meridionale* | NIV | MT237851 |
| YNF16-96 | 2016 | Qujing | *F. meridionale* | NIV | MT237852 |
| YNF16-99 | 2016 | Qujing | *F. meridionale* | NIV | MT237853 |
| YNF16-103 | 2016 | Chuxiong | *F. meridionale* | NIV | MT237854 |
| YNF16-105 | 2016 | Chuxiong | *F. meridionale* | NIV | MT237855 |
| YNF16-106 | 2016 | Chuxiong | *F. meridionale* | NIV | MT237856 |
| YNF16-107 | 2016 | Chuxiong | *F. meridionale* | NIV | MT237857 |
| YNF16-110 | 2016 | Chuxiong | *F. meridionale* | NIV | MT237858 |
| YNF16-112 | 2016 | Honghe | *F. meridionale* | NIV | MT237859 |
| YNF16-117 | 2016 | Kunming | *F. meridionale* | NIV | MT237860 |
| YNF16-119 | 2016 | Kunming | *F. meridionale* | NIV | MT237861 |
| YNF16-122 | 2016 | Kunming | *F. meridionale* | NIV | MT237862 |
| YNF16-123 | 2016 | Kunming | *F. meridionale* | NIV | MT237863 |
| YNF16-125 | 2016 | Kunming | *F. meridionale* | NIV | MT237864 |
| YNF16-22 | 2016 | Zhaotong | *F. cerealis* | NIV | MT237733 |
| YNF15-04 | 2015 | Wenshan | *F.verticillioides* | FUM | MT237865 |
| YNF15-98 | 2015 | Wenshan | *F.verticillioides* | FUM | MT237881 |
| YNF15-39 | 2015 | Wenshan | *F.verticillioides* | FUM | MT237867 |
| YNF16-54 | 2016 | Chuxiong | *F.verticillioides* | FUM | MT237903 |
| YNF15-10 | 2015 | Wenshan | *F. proliferatum* | FUM | MT237866 |
| YNF15-48 | 2015 | Lincang | *F. proliferatum* | FUM | MT237868 |
| YNF16-118 | 2016 | Kunming | *F. proliferatum* | FUM | MT237904 |
| YNF16-127 | 2016 | Kunming | *F. proliferatum* | FUM | MT237905 |
| YNF16-128 | 2016 | Kunming | *F. proliferatum* | FUM | MT237906 |
| YNF16-129 | 2016 | Kunming | *F. proliferatum* | FUM | MT237907 |
| YNF15-63 | 2015 | Lincang | *F. temperatum* | BEA | MT237869 |
| YNF15-67 | 2015 | Lincang | *F. temperatum* | BEA | MT237870 |
| YNF15-76 | 2015 | Lincang | *F. temperatum* | BEA | MT237871 |
| YNF15-77 | 2015 | Lincang | *F. temperatum* | BEA | MT237872 |
| YNF15-81 | 2015 | Lincang | *F. temperatum* | BEA | MT237908 |
| YNF15-82 | 2015 | Lincang | *F. temperatum* | BEA | MT237873 |

*(Continued)*

**Supplementary Table 3 | Continued**

| **Isolate** | **Year** | **Locations** | **Species** | **Toxigenic chemotypes** | **Accession number** |
| --- | --- | --- | --- | --- | --- |
| YNF15-83 | 2015 | Lincang | *F. temperatum* | BEA | MT237874 |
| YNF15-84 | 2015 | Lincang | *F. temperatum* | BEA | MT237875 |
| YNF15-86 | 2015 | Lincang | *F. temperatum* | BEA | MT237876 |
| YNF15-91 | 2015 | Lincang | *F. temperatum* | BEA | MT237877 |
| YNF15-95 | 2015 | Lincang | *F. temperatum* | BEA | MT237878 |
| YNF15-96 | 2015 | Lincang | *F. temperatum* | BEA | MT237879 |
| YNF16-04 | 2016 | Zhaotong | *F. temperatum* | BEA | MT237880 |
| YNF16-08 | 2016 | Zhaotong | *F. temperatum* | BEA | MT237882 |
| YNF16-10 | 2016 | Zhaotong | *F. temperatum* | BEA | MT237883 |
| YNF16-11 | 2016 | Zhaotong | *F. temperatum* | BEA | MT237884 |
| YNF16-12 | 2016 | Zhaotong | *F. temperatum* | BEA | MT237885 |
| YNF16-16 | 2016 | Zhaotong | *F. temperatum* | BEA | MT237886 |
| YNF16-26 | 2016 | Zhaotong | *F. temperatum* | BEA | MT237887 |
| YNF16-32 | 2016 | Zhaotong | *F. temperatum* | BEA | MT237888 |
| YNF16-34 | 2016 | Zhaotong | *F. temperatum* | BEA | MT237889 |
| YNF16-40 | 2016 | Zhaotong | *F. temperatum* | BEA | MT237890 |
| YNF16-42 | 2016 | Zhaotong | *F. temperatum* | BEA | MT237891 |
| YNF16-45 | 2016 | Zhaotong | *F. temperatum* | BEA | MT237892 |
| YNF16-47 | 2016 | Zhaotong | *F. temperatum* | BEA | MT237893 |
| YNF16-48 | 2016 | Zhaotong | *F. temperatum* | BEA | MT237894 |
| YNF16-77 | 2016 | Qujing | *F. temperatum* | BEA | MT237895 |
| YNF16-90 | 2016 | Qujing | *F. temperatum* | BEA | MT237896 |
| YNF16-93 | 2016 | Qujing | *F. temperatum* | BEA | MT237897 |
| YNF16-97 | 2016 | Qujing | *F. temperatum* | BEA | MT237898 |
| YNF16-102 | 2016 | Qujing | *F. temperatum* | BEA | MT237899 |
| YNF16-116 | 2016 | Kunming | *F. temperatum* | BEA | MT237900 |
| YNF16-131 | 2016 | Kunming | *F. temperatum* | BEA | MT237901 |
| YNF16-132 | 2016 | Kunming | *F. temperatum* | BEA | MT237902 |
| YNF15-61 | 2015 | Lincang | *F. incarnatum* | 3-AcDON | MT237909 |
| YNF15-87 | 2015 | Lincang | *F. incarnatum* | 3-AcDON | MT237910 |
| YNF15-93 | 2015 | Lincang | *F. incarnatum* | 15-AcDON | MT237911 |
| YNF16-03 | 2016 | Zhaotong | *F. incarnatum* | 3-AcDON | MT237912 |
| YNF15-01 | 2015 | Wenshan | *F. equiseti* | ZEN | MT237913 |
| YNF15-17 | 2015 | Wenshan | *F. equiseti* | ZEN | MT237914 |
| YNF15-26 | 2015 | Wenshan | *F. equiseti* | ZEN | MT237915 |
| YNF15-27 | 2015 | Wenshan | *F. equiseti* | ZEN | MT237916 |
| YNF15-30 | 2015 | Wenshan | *F. equiseti* | ZEN | MT237917 |
| YNF15-35 | 2015 | Wenshan | *F. equiseti* | ZEN | MT237918 |
| YNF15-38 | 2015 | Wenshan | *F. equiseti* | ZEN | MT237919 |
| YNF15-64 | 2015 | Lincang | *F. equiseti* | ZEN | MT237920 |

*(Continued)*

**Supplementary Table 3 | Continued**

| **Isolate** | **Year** | **Locations** | **Species** | **Toxigenic chemotypes** | **Accession number** |
| --- | --- | --- | --- | --- | --- |
| YNF15-90 | 2015 | Lincang | *F. equiseti* | ZEN | MT237921 |
| YNF16-31 | 2016 | Zhaotong | *F. equiseti* | ZEN | MT237922 |
| YNF16-36 | 2016 | Zhaotong | *F. equiseti* | ZEN | MT237923 |
| YNF16-124 | 2016 | Kunming | *F. equiseti* | ZEN | MT237924 |
| YNF16-15 | 2016 | Zhaotong | *F. avenaceum* | BEA | MT237775 |
| YNF16-14 | 2016 | Zhaotong | *F. avenaceum* | BEA | MT237815 |
